# Construction of Supramolecular Systems That Achieve Lifelike Functions

**DOI:** 10.3390/ma15072391

**Published:** 2022-03-24

**Authors:** Taisuke Banno, Daichi Sawada, Taro Toyota

**Affiliations:** 1 Department of Applied Chemistry, Faculty of Science and Technology, Keio University, 3-14-1 Hiyoshi, Kohoku-ku, Yokohama 223-8522, Japan; banno@applc.keio.ac.jp (T.B.); sawa2744@a3.keio.jp (D.S.); 2 Department of Basic Science, Graduate School of Arts and Sciences, The University of Tokyo, 3-8-1 Komaba, Meguro-ku, Tokyo 153-8902, Japan; 3 Universal Biology Institute, The University of Tokyo, 3-8-1 Komaba, Meguro-ku, Tokyo 153-8902, Japan

**Keywords:** supramolecular chemistry, systems chemistry, non-equilibrium system, amphiphiles, molecular conversions

## Abstract

The Nobel Prize in Chemistry was awarded in 1987 and 2016 for research in supramolecular chemistry on the “development and use of molecules with structure-specific interactions of high selectivity” and the “design and production of molecular machines”, respectively. This confirmed the explosive development of supramolecular chemistry. In addition, attempts have been made in systems chemistry to embody the complex functions of living organisms as artificial non-equilibrium chemical systems, which have not received much attention in supramolecular chemistry. In this review, we explain recent developments in supramolecular chemistry through four categories: stimuli-responsiveness, time evolution, dissipative self-assembly, and hierarchical expression of functions. We discuss the development of non-equilibrium supramolecular systems, including the use of molecules with precisely designed properties, to achieve functions found in life as a hierarchical chemical system.

## 1. Introduction

The existence of strong forces acting between specific molecules and ions (intermolecular interactions) began to be recognized after Pedersen serendipitously discovered the unique physical properties of cyclic ethers (crown ethers) in 1967 [1]. When Pedersen, Lehn, and Cram were awarded the Nobel Prize in Chemistry (1987) for their “achievements in the development of molecules with structure-specific selectivity” [2], the development of structures and their functions, expressed by the structure-selective self-assembly of molecules through specific intermolecular interactions (molecular recognition) became extremely popular. Subsequently, the foundation of supramolecular chemistry was established. In the 21st century, the natural sciences have progressed from equilibrium to non-equilibrium systems (Prigogine was awarded the Nobel Prize in Chemistry in 1977 for the concept of dissipative structures in non-equilibrium systems [3]), from static to dynamic systems (Sauvage, Stoddart, and Feringa were awarded the Nobel Prize in Chemistry in 2016 for the construction of molecular machines [4]), and from simple systems to complicated systems (the Nobel Prize in Physics was awarded in 2021 for the contributions of statistical mechanics to dynamic and complex systems [5]). Molecular assemblies in supramolecular chemistry that evolve over time in non-equilibrium systems, such as the formation of thermodynamically stable structures and the dynamics of self-assembled aggregates involving non-equilibrium systems and non-linear reactions, have been developed in the field of systems chemistry [6]. This review explains the systems chemistry of supramolecular structures in non-equilibrium systems using designed and synthesized organic molecules. Unlike biopolymers, such as proteins, RNA, and DNA, these relatively simple organic molecules can be synthesized based on a molecular design that carefully assumes specific and multipoint intermolecular interactions at the molecular level. The reason for this is that it enables the analysis of the dynamics of complicated and hierarchical molecular self-assembly to better understand the characteristics and properties of non-equilibrium systems.

First, we introduce the response of supramolecular structures to stimuli. The gradual change in a system (molecular assembly) from an initial state to the final state by applying a stimulus (i.e., suddenly changing the environment) is a process of transitioning from one equilibrium state to the next (or steady state) and can be considered as a relaxation phenomenon. This simple non-equilibrium state has attracted interest since the early days of supramolecular chemistry as a function of the stimulus response. Second, we describe a time evolution system in which the supramolecular structure changes as the constituent molecules undergo molecular transformation during self-assembly. If the system is in equilibrium between the assembly and the monomer, the supramolecular structure may change via the monomer. Based on this compositional change, a system of supramolecular structures can exhibit a transition or crossover. Third, we explain that non-equilibrium systems involving multiple chemical reactions and chemical fuels (or sacrificial reagents) are present. Thus, behaviors such as the transient formation of supramolecular structures, spontaneous changes in size, and driving from dissipative processes appear. Finally, we will present examples of studies that involve inducing the self-propulsion of supramolecular structures and communication between structures using hierarchical chemical systems triggered by chemical reactions or by incorporating cascade reactions. These hierarchical systems are expected to result in the construction of lifelike supramolecular systems (e.g., self-reproduction, chemotaxis, and environmental adaptation).

## 2. Response of Supramolecular Self-Assembly to Stimuli

There is a vast array of precise and efficient self-assembly processes involved in the fundamental role of DNA plays in living systems with its replication and translation functions. To mimic the functions of such biomolecules using simple synthetic molecules, supramolecular materials with functions that can be switched on and off in response to stimuli have been created. Here, we present examples of how the physical properties of supramolecular materials can be changed by external stimuli such as the preparation conditions of the molecular assembly, pH changes, and light irradiation. In particular, we explain the properties that have been achieved by designing the structure of molecules to enable them to be used as shape memory materials and storage and transport carriers (that is, gels and vesicles).

### 2.1. Gels

**Figure 1:**
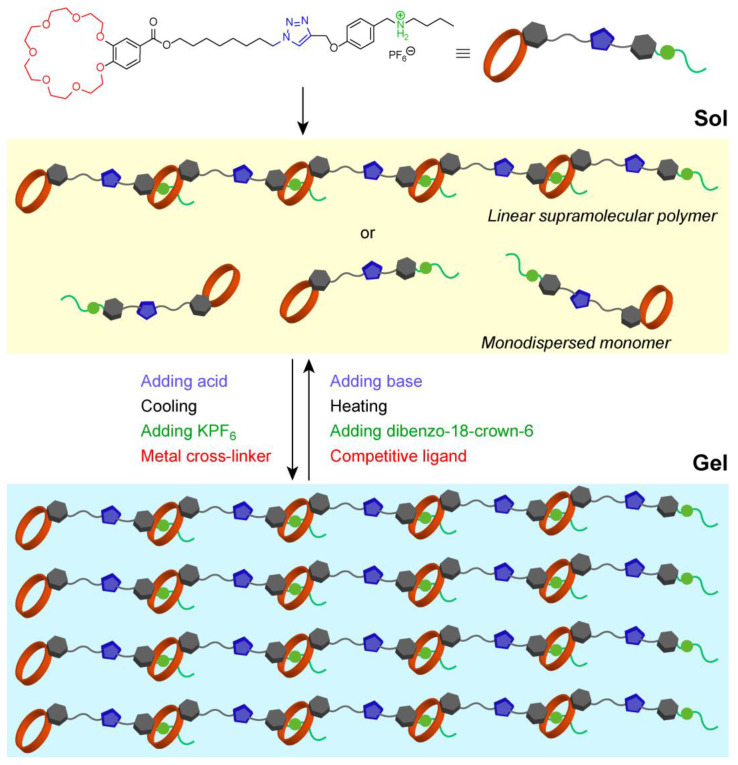
Schematic illustration of sol-gel transition from the collapse and formation of supramolecular polymers in response to pH, heat, cationic species, and metal ions.

There have been many reports on supramolecular gels since their physical properties can be measured using a variety of methods, and their formation can be confirmed by the simple method of inversion in a test tube. Van Esch and Feringa suggested that supramolecular gels can be created based on strategic molecular design principles. Many supramolecular gels have been developed based on original molecular design guidelines for organic chemists [7]. In the 1990s, supramolecular gels that respond to a single stimulus were developed. These include peptides that form gels depending on the polarity of the solvent and pH [8]. In addition, a reversible transition between the gel and sol states in response to light irradiation has been reported [9]. The structure and stability of the gel can be controlled by host-guest interactions [10,11]. In the 2000s, supramolecular gels that form mediated by halogen bonds [12] and those containing >95% water, so-called ‘aqua materials’ [13] were developed by controlling intermolecular forces such as electrostatic and hydrophobic interactions based on the molecular structure. Furthermore, supramolecular gels that respond to multiple stimuli and enable the precise control of their physical properties have also been developed. For example, Huang and co-workers reported that molecules with crown ethers and secondary amine salts at both ends could be used in high concentrations to produce linear supramolecular polymer gels. Furthermore, when Pd ions were added to the gels, the supramolecular polymers cross-linked to form a network structure with excellent mechanical strength. These gels also undergo a sol-gel transition from the collapse and formation of supramolecular polymers caused by pH, heat, cationic species, and metal ions (Figure 1) [14]. Supramolecular gels that form in response to multiple stimuli such as temperature changes, light irradiation, pH, and stress have also been developed [15,16,17]. Supramolecular polymers that respond to multiple stimuli have promising applications as materials with shape memory and self-healing abilities.

**Figure 2:**
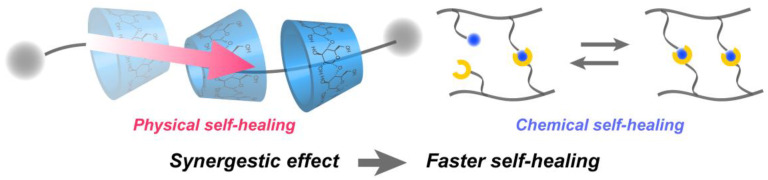
Schematic illustration of the fast self-healing of supramolecular gel by the synergistic effect.

In particular, the self-healing ability (that is, the ability to spontaneously self-repair after being damaged) is an emergent function that appears through the skillful control of intermolecular interactions and is applied to various chemical products and medical materials [18,19]. The main gels developed thus far are those mediated by bonds that reconnect even if broken (that is, ionic bonds [20,21,22], coordinate bonds [23,24,25], hydrogen bonds [26,27,28], and host-guest interactions [29,30,31]). However, there is a trade-off between the mechanical strength and self-healing ability, as harder materials are difficult to repair when damaged. Harada and co-workers solved this problem using polyrotaxanes, which have a necklace-like structure with string-like molecules penetrating ring-like molecules, as a platform to connect the ring molecules of cyclodextrins using dynamic covalent bonds between boronic acid and diol (Figure 2) [32]. The synergistic effect of the mobility of cyclodextrin in polyrotaxane, which enables it to move freely on the string (called physical self-healing), and the reversible nature of dynamic covalent bonds (called chemical self-healing) results in a considerably increased rate of self-healing of the supramolecular gel. It should be noted that the gel was as stiff as a supramolecular gel possessing only one of these two properties. Recently, the same group reported that mixing polymers with cyclodextrin (host) and adamantane (guest) by ball milling resulted in a gel that exhibited faster rate of self-healing than those of gels prepared by casting with solvent volatilization [33].

### 2.2. Vesicles

The molecular self-assembly of surfactants and other amphiphilic molecules can be explained by intermolecular interactions based on the critical packing parameter as explained precisely by Israelachvili [34]. Among these, vesicles, which have a closed capsule structure of bilayers formed by amphiphilic molecules in water, have been used in various fields, including drug delivery systems, since their unique structure enables them to hold both hydrophilic and hydrophobic compounds. The conditions for vesicle formation using synthesized molecules have been thoroughly investigated. Kunitake et al. demonstrated that synthetic amphiphiles such as didodecyldimethylammonium bromide can form vesicles in water [35]. Since then, research has been conducted to synthesize amphiphiles based on precise molecular design strategies and to clarify the relationship between their molecular structure and function. For example, molecular assemblies comprising cationic and anionic surfactants form vesicles at specific compositions and above a certain temperature [36,37]. Tameyuki et al. reported that vesicles containing cationic amphiphilic molecules with amide bonds, which are formed after thermal stimulation, change their morphology in response to subsequent temperature changes [38]. They revealed that, although oleic acid spontaneously formed vesicles under basic conditions [39,40] when it coexisted with didodecyldimethylammonium bromide, the pH range for vesicle formation slightly increased [41]. Sawada et al. showed that vesicles comprising cationic amphiphilic molecules with imine bonds and oleic acid are stable over a wide pH range from basic to acidic values [42]. Vesicles formed by amphiphilic polymers (called polymersomes) containing carboxylic acid moieties, such as methacrylic acid and acrylic acid, also form vesicles under acidic conditions below pH 4 [43,44]. Van Hest and co-workers reported that polymersomes formed by block copolymers of polyethylene glycol and poly(_D,L_-lactic acid) have different film thicknesses and can deform into stomatocytes (invaginated vesicles) and tubular vesicles, depending on both the composition and solvent used during preparation [45,46]. This may be due to the interaction between polyethylene glycol and poly(_D,L_-lactic acid) in the polymer chain and solvent molecules. This is also a very interesting example of how subtle differences in the chemical composition can influence the self-assembly process.

**Figure 3:**
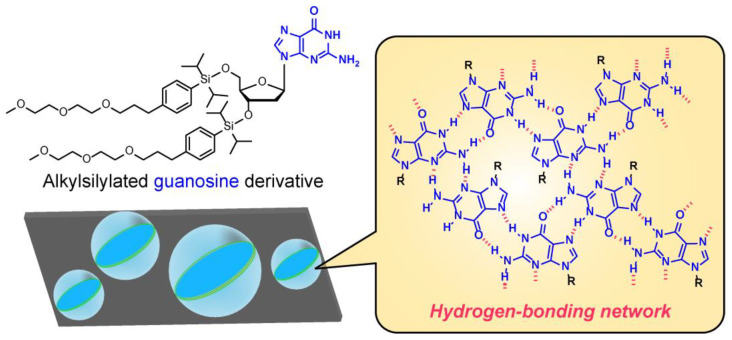
Schematic illustration of the hydrogen-bonding network of guanosine derivatives with alkyl silyl groups that results in the stable vesicles.

Many attempts have been made to add additional functions to vesicles by focusing on intermolecular interactions. For example, Sakaino et al. synthesized guanosine derivatives with alkyl silyl hydrophobic groups. They showed that vesicles composed of these derivatives retain their membrane structure in a water-free vacuum and are so strong that they can be injected with chemicals through the insertion of glass capillaries [47]. This is presumably due to the formation of a guanosine-derived hydrogen-bonding network in the vesicle membrane (Figure 3). Muraoka et al. developed an amphiphilic molecule that mimics transmembrane proteins in biological membranes. It acts as an ion channel when folded and assembled in a vesicle membrane [48]. The same research group found that when its derivative and chemical ligand form a complex, pores are created in the vesicle membrane [49]. When cyclodextrin was added to the pores, the pores closed as the complex collapsed due to the inclusion of the ligand. The introduction of such transmembrane protein-like artificial molecules into vesicular membranes only enables the uptake/release of chemicals from the external or internal region under certain conditions at pH 8 [50]. Furthermore, polymersomes exhibit better structural stability than those formed by low molecular weight amphiphilic molecules. In particular, nanometer-sized polymersomes have different shapes depending on the monomer composition in the polymer, which results in different affinities for living cells [45,51,52]. These findings have important implications for nanomedicine.

### 2.3. Other Supramolecular Self-Assemblies

Although there have been many reports on micelles, which are spherical self-assemblies formed by amphiphilic molecules in water, here we introduce micelles that develop through chemical reactions. Imine bonds, which are dynamic covalent bonds, are often used to fabricate functional materials due to their reversibility in which the dehydration/hydrolysis reactions proceed at a specific pH. Zhang and co-workers reported that, in a mixture of methoxy-(polyethylene glycol)-b-poly(lysine hydrochloride) and lipophilic aldehyde, the dehydration reaction proceeds at pH 7.4 to form imines, resulting in polymer micelles [53]. At pH 6.5, hydrolysis occurred and the amine portion of lysine was neutralized, resulting in the collapse of the micelles. This indicates that the switch between the uptake and release of chemicals can be performed within a very narrow pH range. Therefore, this type of micelle has the potential to be an intelligent material. Giuseppone and co-workers reported that aldehydes, which are components of fragrances, attach to the terminal amine of polyethylene glycol-polyphenylene oxide surfactants to form micelles and that the fragrance components are released from the micelles when hydrolysis proceeds under specific pH conditions [54]. When various oil components were solubilized as fragrances, the more volatile oil components were more easily retained inside the micelles, and evaporation of the less volatile components was accelerated [55]. This is an interesting example of how the balance of release of solubilized fragrance components can be controlled. Thus, the concept of dynamic covalent bonding is very useful for creating materials whose functions are expressed under specific conditions.

## 3. Time Evolution of Supramolecular Self-Assemblies

One of the characteristics of living systems is that they form an ordered structure through the assembly and disassembly of many component molecules that develop autonomously while becoming a higher-order structure depending on the environment. Attempts to artificially induce such phenomena involving time evolution in structures could be an important tool for understanding how molecules self-assemble to achieve higher-order functions. Toyota et al. reported a phenomenon involving time evolution in which surfactant molecules with imine bonds hydrolyze to form hydrophilic quaternary ammonium salts and lipophilic amines over time, resulting in the transformation of micelles into worm-like micelles, plain lamellae, and giant vesicles that then grow and expand into their nested forms [56]. A continuous phase transition has also been reported, in which the Huisgen cycloaddition reaction between hydrophilic azides and lipophilic alkynes proceeds to change the oil-droplet-dispersed solution to a micellar solution, and the resulting triazole and Pd ions formed a complex and led to gelation in the presence of different Cu and Pd ions [57]. Furthermore, different transitions of supramolecular self-assemblies were observed depending on the order of photoisomerization of the azobenzene skeletons and reduction of the disulfide groups [58]. This pathway-dependent transition provides a new concept for developing stimuli-responsive materials that exhibit desirable properties in specific environments. In this section, we introduce some examples of lifelike phenomena involving time evolution that can be created using functional molecules synthesized based on precise molecular design strategies.

### 3.1. Supramolecular Polymerization

Polymerization is typically an organic reaction in which monomers are connected by covalent bonds to form polymers. Supramolecular insights are also important when considering the properties of the resulting polymers. For example, the toughness of synthetic nylons originates from the hydrogen bonds between the polymer chains. In addition, supramolecular polymers exhibit dynamic properties such as directionality and reversibility. It is widely known that the formation of supramolecular polymers can be revealed from their molecular properties by carefully examining their intermolecular interactions.

The concept of supramolecular polymerization was introduced by Meijer and co-workers [59]. Using π-conjugated molecules with hydrogen-bonding units at their terminals, they successfully used spectroscopic measurements to monitor nucleation during the formation of a helical fibril structure via self-assembly [60]. By investigating the process in more detail, they were able to clarify the process by which the structure grew [61]. The self-assembly of the building blocks in nonpolar solutions was initiated by the formation of dimers via quadruple hydrogen bonds. This dimer further self-assembled to form a helical stack via a nucleation-elongation mechanism. In this process, although the kinetically favorable right-handed P-helix structure was formed quickly, it was less stable than the slowly forming left-handed M-helix structure, and only the P-helix structure could be obtained.

**Figure 4:**
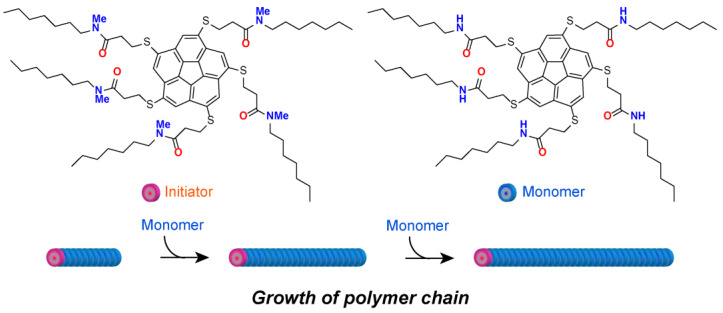
Schematic illustration for supramolecular polymerization of corannulene derivatives having five amide-adducted thioalkyl side chains.

Manners and co-workers found that when an additional polymer was added to polymer-forming cylindrical micelles, the micelles were epitaxially elongated with a very narrow size distribution [62,63]. This behavior is similar to that observed in living polymerization. Sugiyasu and co-workers, inspired by prion infections, reported an ‘artificial infection’ process in which monomers of the porphyrin skeleton assembled to form nanoparticles, which further grew in the presence of a fixed number of nanofibers that acted as ‘pathogens’ [64]. These are all living supramolecular polymerization systems that do not require an initiator since they are sequential polymerizations. In contrast, for the first time, Aida and co-workers reported a chain-growth supramolecular polymerization system using a corannulene derivative with five amide-adducted thioalkyl side chains as the initiator and monomer (Figure 4) [65]. In low-polarity solvents, the monomer molecules did not polymerize since they formed a bowl from intramolecular hydrogen bonds between C=O in one chain and NH in another chain. When the *N*-methylated initiator of the monomer molecule was added as a proton acceptor, hydrogen bonds were reconstituted between the initiator and monomer to form active species. Subsequently, polymerization was initiated, and the monomers were added to the chain. Furthermore, introducing chirality into the side chains of the initiator and monomer revealed that the polymerization could proceed with the same stereochemistry between the molecules. Sugiyasu and co-workers reported a system in which the polymerization behavior can be controlled using light to switch the activity/inactivity of dormant species [66]. Recently, there have been reports of systems in which the steric structure of polymers can be controlled via stereoselective seed growth [67]. There have also been reports of supramolecular polymerization systems in which the properties of the liquid crystal produced by the polymerization process differ depending on the temperature conditions [68], thus enabling more precise control of the physical properties of the resulting structure.

### 3.2. Polymerization-Induced Self-Assembly (PISA)

The use of a living polymerization reaction as a system in which structures change over time is very useful for elucidating the relationship between the reaction and the structural transition, as well as for gradually changing the properties of the constituent components. Armes et al. proposed the concept of polymerization-induced self-assembly (PISA) [69,70], in which the reversible addition fragmentation chain transfer (RAFT) polymerization reaction, one of the living reactions, proceeds in an aqueous medium and results in a continuous transition of the structure as the hydrophobic moieties in the amphiphilic polymer elongate. In particular, the use of poly(2-(methacryloyloxy)ethylphosphorylcholine) (PMPC) and poly(glycerol monomethacrylate) as hydrophilic groups to polymerize lipophilic 2-hydroxypropyl methacrylate results in a gradual morphological change from spherical micelles to strings and then to vesicles via a jellyfish-like shape. Since the polymerization in these systems exhibits living characteristics, the morphology of the nanostructure can be precisely controlled by changing the reaction time. The introduction of host-guest interactions, such as those involving cyclodextrin and lipophilic monomers, into PISA facilitates tight control of the shape of the nanostructures [71,72]. A study investigating the relationship between morphological changes and physical properties has also been conducted. For example, Cao et al. added curcumin as a model drug to PISA, thereby polymerizing methyl methacrylate into PMPC, and studied drug release in PISA by showing the relationship between the morphological change and the amount of curcumin stored in a phase diagram [73]. In addition to RAFT, PISA involving atom transfer radical polymerization (ATRP), nitroxide-mediated polymerization, ring-opening polymerization, and living anionic polymerization has also been reported [74]. Recently, polymerization-induced electrostatic self-assembly, in which PISA of a cationic polymer proceeds in the presence of an anionic polymer, has been reported [75]. The morphology of the structure, as well as the temperature response, varied depending on the degree of polymerization. Polysaccharide-based PISA has also been reported for biomedical applications [76]. Since many PISAs eventually result in the formation of vesicles, this reaction system may be considered a model for compartmentalization in biogenesis [77]. In addition, simultaneous enlargement/growth and disintegration of vesicles [78], as well as size changes and self-propulsion have been observed in a PISA system [79]. Consequently, PISA has attracted significant attention from researchers of primitive cell models.

### 3.3. Dynamic Combinatorial Chemistry (DCC)

Combinatorial chemistry is a method for searching for compounds that exhibit the desired functions and properties of many compounds synthesized simultaneously. It has mainly been used for the development of drugs, along with automation in organic synthesis. The structures and proportions of the generated molecular assemblies differ over time based on the chemical composition of the hydrolysis/dehydration reactions of imines and oxidation/reduction reactions of thiols and disulfides. In such a dynamic combinatorial library (DCL) system, key compounds are autocatalytically generated. Therefore, by coupling DCC with the autocatalytic formation of supramolecular self-assemblies, a system with inherent self-replication can be constructed, which is an essential feature of life. Giuseppone and co-workers reported that the reaction of aldehydes with amines results in surfactant components with imine bonds, which leads to the self-growth of micelles [80]; van Esch and co-workers reported similar micellar systems independently [81]. A chemical system that transitions between isotropic and nematic phases has also been reported [82]. The transition occurs as the chemical composition changes based on the thermodynamic equilibrium in a constitutional dynamic library when an electric field is applied to a liquid sample containing imines and amines.

**Figure 5:**
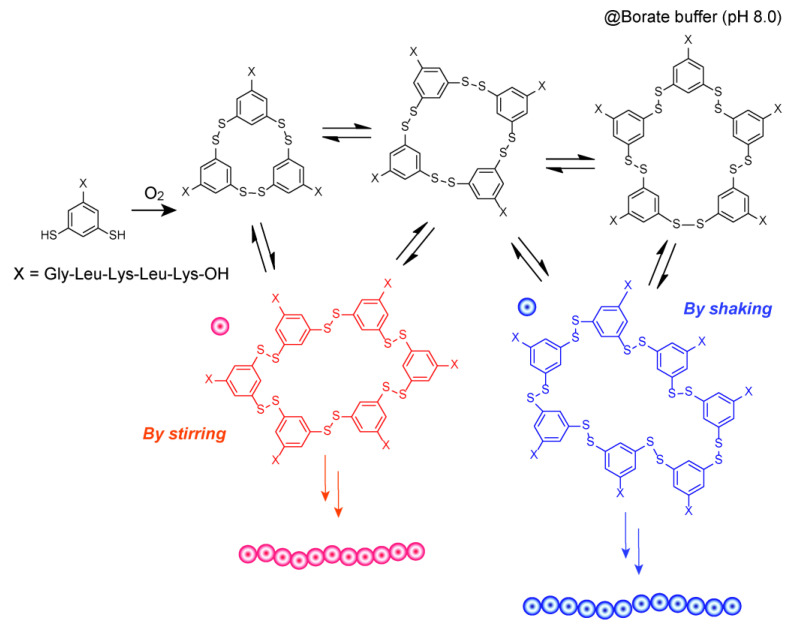
Schematic illustration of production of various macrocycles and elongation of fibers composed of hexamers and heptamers when dithiols with peptide chains were oxidized with stirring or shaking.

Otto and co-workers reported a variety of interesting chemical systems using thiol-disulfide conversion. When dithiols with peptide chains were oxidized upon mechanical stimuli such as stirring or shaking, various peptide-derived macrocycles were produced in the DCL (Figure 5) [83]. Cyclic hexamers and heptamers gradually became the main products under shaken and stirred conditions, respectively. Cryogenic transmission electron microscopy images showed that the hexamers and heptamers formed elongated fibers with diameters of ~1 nm, which roughly corresponded to the length of a single ring structure. Since the β-sheet structure was confirmed in the circular dichroism spectrum, the fiber was thought to have been formed by unidirectional stacking of the rings. In a mixture of borate buffer and dimethylformamide, the use of oxyethylene chains instead of peptide sequences resulted in the formation of various cyclic disulfides of up to 51 monomers under unstirred conditions. However, only cyclic hexamers were formed at thermodynamic equilibrium under stirred conditions. This affords thermodynamically stable nanoribbon structures. Trimers and tetramers are considered to be stable structures since they can form rings. Nevertheless, cyclic hexamers were predicted to be the main product since stirred conditions enable autocatalytic pathways, which lower the activation energy barrier for hexamer formation [84]. In DCLs with similar compounds, the formation of various membrane structures, including vesicles and sponge phases, is accelerated by the formation of their constituents [85]. Since a large number of aggregates are formed in this system by the generation of macrocyclic compounds with various hydrophilic/hydrophobic properties due to the oxidation of building blocks, it is useful as a DCC to carefully examine the requirements for compartmentalization among the aggregates. This group also successfully constructed a chemical system in which the formation of disulfides from thiols was autocatalytically promoted by the binding of cofactors under light irradiation, resulting in the self-replication of cyclic hexamers [86]. Furthermore, they constructed a system in which a replicant influences the emergence of another replica [87]. These cases are interesting since they embody the selection pressure of molecules and their self-assemblies under perturbations of the system, and they provide important insights into the origin-of-life model in molecular science.

## 4. Dissipative Self-Assembly 

**Figure 6:**
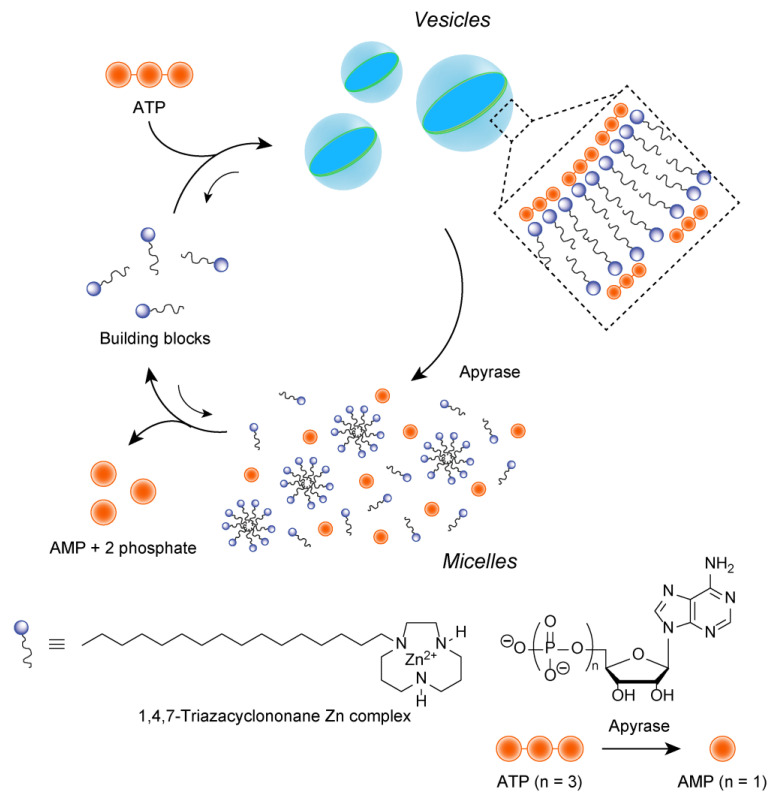
Schematic illustration of a vesicle-micelle transformation cycle coupled with consuming ATP. Apyrase is one of ATP-degrading enzymes.

The presence of chemical fuels (or sacrificial reagents) has been reported to cause the transient formation of supramolecular self-assemblies or spontaneous morphological changes in systems that incorporate multiple chemical reactions. Prins and co-workers used molecular recognition between the zinc complex of 1,4,7-triazacyclonane and the phosphate moiety of adenosine triphosphate (ATP) to design and synthesize a new amphiphilic molecule with the zinc complex of 1,4,7-triazacyclononane as the hydrophilic part (Figure 6) [88]. This molecule formed micelles in an aqueous solution, but when ATP was added, the micelles became vesicles that were several tens of nanometers in size. When ATP-degrading enzymes were added to the system to play a dissipative role prior to the formation of a transient structure, the vesicles were broken down as adenosine monophosphate (AMP) was formed, and the amphiphilic molecules formed their original micelles. When ATP was added to this system, vesicles formed again, but they were gradually converted to micelles. This group also designed and synthesized an amphiphilic molecule with the zinc complex of 1,4,7-triazacyclononane as the hydrophilic part, noting that the zinc complex of 1,4,7,10-tetraazacyclododecane recognizes the phosphate moiety of monophosphate nucleic acids such as AMP [89]. They also reported transient formation of vesicles from micelles in the presence of monophosphate nucleic acid-hydrolyzing enzymes. In this case, molecular recognition between the amphiphilic molecules of the zinc complex and the monophosphate nucleic acid showed that the vesicles were only temporally formed in certain combinations. This indicates that flexible design by molecular structure may be possible in the dissipative system of upregulation in transient structure formation.

Derivatives of oligopeptides with specific amino acid sequences self-assemble into fibrous structures that are insoluble in water. Prior to transient formation of this structure, Tena-Solsona et al. attempted the dehydration condensation of fatty acids with dipeptides in water, using a derivative of carbodiimide as fuel [90]. The anhydride produced by dehydration condensation formed a network of fiber-shaped structures, but hydrolysis predominated over time, and the structure disappeared. Kumar et al. constructed a reaction system for the binding of amino acids to naphthalimides with monoamino acids and an acylated C-terminus in the presence of enzymes [91]. This enzyme can also hydrolyze the acylated C-terminus. The building blocks that became dipeptides self-assembled to form gels, but after an induction period, hydrolysis became dominant, and sols were formed. It should be noted that, although the fibrous structures were only a few nanometers thick, they could temporally form structures a few millimeters in size while consuming chemical fuel during the dissipation process. The fact that the fibrous structures form a network is crucial for this transition. Furthermore, the process (particularly the lifetime of the structure) can be controlled by its molecular structure.

The self-propulsion phenomenon of supramolecular structures can also be observed during the dissipation process using chemical fuels. Although many mechanisms are known to propel objects comprising chemical substances, platinum nanoparticles (PtNPs) have attracted considerable attention in terms of self-assembly [92]. PtNPs are self-assembled by reducing platinum chloride, and their size and shape are controlled by coexisting substances. The PtNPs are propelled by a driving force when they decompose hydrogen peroxide to produce oxygen [93]. Hayakawa et al. encapsulated PtNPs in micrometer-sized anisotropic hydrogels and controlled the self-propulsion of these hydrogels [94]. The research group of van Hest and Wilson developed anisotropic polymersomes that encapsulate PtNPs in the stomatocytes of the polymersomes, resulting in an enhanced unidirectional self-propulsion system [95]. They introduced poly(*N*-isopropylacrylamide) [96], an inclusion of azobenzene and cyclodextrin [97], and a complex of α-poly-_L_-lysine and ATP [98] into the polymersomes and showed that their stimuli responsiveness can be used to modulate the state of self-propulsion.

Oil and liquid crystal droplets have also been used in self-propulsion systems for molecular aggregates in water respectively. The chemical Marangoni effect is the driving force in many of these systems [99]. Among these, systems designed to maintain a continuous supply of fuel for propulsion from outside the oil droplets and the continuous consumption of fuel have been reported by Toyota et al. and Babu et al. independently. Toyota et al. dispersed oil droplets of 4-octylaniline in an aqueous solution of cationic amphiphilic molecules containing imine bonds and observed that oil droplets containing an acid catalyst were propelled in one direction while producing many oily particles on the rear side [100]. Badu et al. reported that when 1-hexanethiol and water-soluble 2-methacryloyloxyethyl phosphorylcholine were added to octanol droplets, they combined with an addition reaction to form an amphiphilic molecule, and the chemisorption of the amphiphilic molecule propelled the octanol droplets by the Marangoni effect [101]. Interestingly, the moving octanol droplets accelerated this addition reaction, indicating that the consumption of chemical fuel formed in a positive feedback loop with the propulsion of the droplets.

## 5. Functionalization of Supramolecular Self-Assemblies with Hierarchical Properties

Finally, we introduce higher-order systems for the self-propulsion of supramolecular structures and communication between supramolecular self-assemblies. These are constructed using a hierarchical chemical reaction system triggered by the incorporation of a cascade of chemical reactions. The formation of spatially higher-order structures requires the incorporation of long-range interactions into system design. One long-range interactions is the use of advection. The Marangoni effect plays an important role in controlling this phenomenon through chemical reactions. For example, Nguindjel et al. reported that when protonation of oleate was induced at the water surface using 1-(2-nitrophenyl)ethyl sulfate, which becomes an acid under light irradiation, molecular aggregates are formed and enlarged at the light-irradiated water surface [102]. This indicates that that the oleate and oleic acid were assembled in the light-irradiated region through the Marangoni effect generated by the surface tension gradient.

Furthermore, many attempts have been made to make the dynamics of self-propelled oil droplets more complex. Banno et al. used an aqueous solution of surfactants that was converted into constituent molecules of oil droplets when hydrolyzed by an acid catalyst [103]. They observed that the oil droplets were propelled in an aqueous solution of the surfactant under acidic conditions, but then stopped and split, and the split oil droplets resumed self-propulsion. Oil droplets that exhibit such complex movements are often composed of multiple components [104,105]. Cronin and co-workers correlated the components of self-propelled oil droplets with the characteristic dynamics of droplets on the water surface [106,107,108]. They repeatedly tracked each oil droplet, extracted its components, examined the component ratio, and observed the dynamics of oil droplets while gradually changing the component ratios. Thus, directional evolution of oil droplets with the desired behavior was conducted in the laboratory. This series of studies revealed that the adaptability of oil droplet dynamics can be estimated using information on the component ratio, even without biologically derived signaling molecules such as DNA, RNA, or proteins.

**Figure 7:**
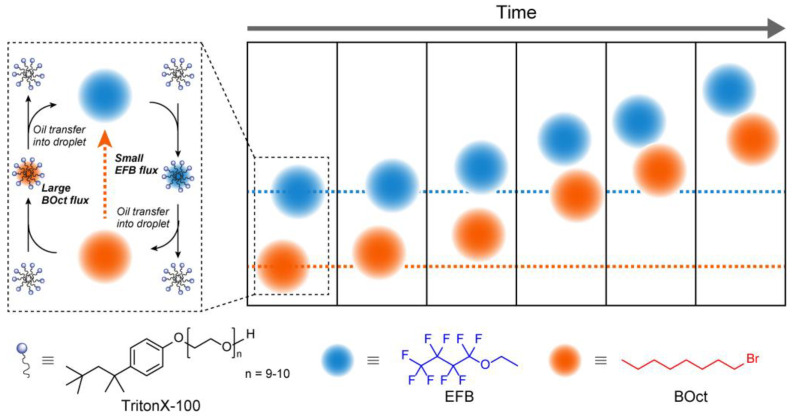
Schematic illustration of a self-propelled oil droplet, 1-bromooctane, chasing a different self-propelled oil droplet, ethoxynonafluorobutane, in a Triton X-100 aqueous solution.

In addition, self-propelled oil droplets can be grouped by the interactions between chemicals. Zarzar and co-workers found that oil droplets with two different components, 1-bromooctane (BOct) and ethoxynonafluorobutane (EFB), self-propelled in aqueous solutions of TritonX-100 (Figure 7) [109]. Furthermore, they found that when these oil droplets coexisted, the BOct oil droplets followed the EFB oil droplet. This indicates that EFB and BOct were slightly emulsified from the oil droplets by TritonX-100, which depended on the spatial field where they were incorporated into each other’s oil droplets. In addition, when the EFB and BOct oil droplets were grouped together and self-propelled, the pattern of movement of the group depended on the number and spatial arrangement of the oil droplets. The formation of a dynamic pattern between self-propelled particles under chemotaxis-dominated conditions is an important result. This suggests that the collective behavior of active matter is governed by simple rules, even without higher-order biological secretion or chemoreception mechanisms.

Moreover, Mason et al. has reported the realization of chemical communication of water-soluble compounds, H_2_O_2_, between droplets of liquid-liquid phase separation (coacervation) in aqueous media [110]. Coacervation is a phenomenon in which droplets are spontaneously generated by intermolecular interactions, such as hydrophobic interaction and electrostatic attraction among macromolecular components. Oparin proposed that spontaneously formed coacervate droplets are structures close to the origin of life. Since then, many primitive cell models including coacervate droplets have been reported [111,112].

## 6. Summary

In this review, we discuss new properties and dynamics of supramolecular self-assemblies, such as stimulus response, time evolution, dissipative structural changes, and the incorporation of nonlinear reactions at the molecular level. Incorporating such reaction systems into supramolecular self-assemblies will enable the induction of lifelike dynamics in systems far from equilibrium, which is one of the significant goals of systems chemistry [113]. Of course, long-term lifelike functions such as evolution, memory and learning, and immunological systems are still beyond its goals. Furthermore, it will also contribute to the development of new materials with higher-order functions improvement of our molecular understanding of the origin of life.

If non-equilibrium systems of supramolecular self-assemblies can be designed and experimentally created from molecular structures, it will be easier to control the functions expressed as outputs. This will also lead to a better understanding of the complex functions observed in life. Currently, the development process is based on a trial-and-error approach, in which the optimal structure is sought by synthesizing derivatives with slightly different molecular structures and evaluating their properties and functions. Recently, it has become possible to analyze the physical properties of molecular assemblies such as vesicles and liquid crystals using molecular simulations and to use the training data collected from these simulations to conduct machine learning to predict the physical properties of molecular self-assemblies based on their molecular structures. If a cycle of predicting the properties of molecules, incorporating them into molecular designs, synthesizing them, and evaluating the functions of the resulting molecular assemblies can be established, strategies for rational molecular design that are supported by theory can be implemented in systems chemistry. Such materials informatics methods are expected to become the core processes for the development of functional materials in near future.

## Data Availability

Not applicable.
